# Siamese neural networks for continuous disease severity evaluation and change detection in medical imaging

**DOI:** 10.1038/s41746-020-0255-1

**Published:** 2020-03-26

**Authors:** Matthew D. Li, Ken Chang, Ben Bearce, Connie Y. Chang, Ambrose J. Huang, J. Peter Campbell, James M. Brown, Praveer Singh, Katharina V. Hoebel, Deniz Erdoğmuş, Stratis Ioannidis, William E. Palmer, Michael F. Chiang, Jayashree Kalpathy-Cramer

**Affiliations:** 1 Athinoula A. Martinos Center for Biomedical Imaging, Department of Radiology, Massachusetts General Hospital, Boston, MA USA; 2 Division of Musculoskeletal Imaging and Intervention, Department of Radiology, Massachusetts General Hospital, Boston, MA USA; 3 Department of Ophthalmology, Casey Eye Institute, Oregon Health & Science University, Portland, OR USA; 4 School of Computer Science, University of Lincoln, Lincoln, UK; 5 Department of Electrical and Computer Engineering, Northeastern University, Boston, MA USA; 6 Department of Medical Informatics and Clinical Epidemiology, Oregon Health & Science University, Portland, OR USA; 7 MGH and BWH Center for Clinical Data Science, Massachusetts General Hospital, Boston, MA USA

**Keywords:** Diagnosis, Machine learning, Medical imaging, Medical research

## Abstract

Using medical images to evaluate disease severity and change over time is a routine and important task in clinical decision making. Grading systems are often used, but are unreliable as domain experts disagree on disease severity category thresholds. These discrete categories also do not reflect the underlying continuous spectrum of disease severity. To address these issues, we developed a convolutional Siamese neural network approach to evaluate disease severity at single time points and change between longitudinal patient visits on a continuous spectrum. We demonstrate this in two medical imaging domains: retinopathy of prematurity (ROP) in retinal photographs and osteoarthritis in knee radiographs. Our patient cohorts consist of 4861 images from 870 patients in the Imaging and Informatics in Retinopathy of Prematurity (i-ROP) cohort study and 10,012 images from 3021 patients in the Multicenter Osteoarthritis Study (MOST), both of which feature longitudinal imaging data. Multiple expert clinician raters ranked 100 retinal images and 100 knee radiographs from excluded test sets for severity of ROP and osteoarthritis, respectively. The Siamese neural network output for each image in comparison to a pool of normal reference images correlates with disease severity rank (*ρ* = 0.87 for ROP and *ρ* = 0.89 for osteoarthritis), both within and between the clinical grading categories. Thus, this output can represent the continuous spectrum of disease severity at any single time point. The difference in these outputs can be used to show change over time. Alternatively, paired images from the same patient at two time points can be directly compared using the Siamese neural network, resulting in an additional continuous measure of change between images. Importantly, our approach does not require manual localization of the pathology of interest and requires only a binary label for training (same versus different). The location of disease and site of change detected by the algorithm can be visualized using an occlusion sensitivity map-based approach. For a longitudinal binary change detection task, our Siamese neural networks achieve test set receiving operator characteristic area under the curves (AUCs) of up to 0.90 in evaluating ROP or knee osteoarthritis change, depending on the change detection strategy. The overall performance on this binary task is similar compared to a conventional convolutional deep-neural network trained for multi-class classification. Our results demonstrate that convolutional Siamese neural networks can be a powerful tool for evaluating the continuous spectrum of disease severity and change in medical imaging.

## Introduction

The evaluation of disease severity and change over time on medical images are important and routine tasks. For example, vascular changes in retinopathy of prematurity (ROP) can be an important biomarker of disease progression and response to therapy^1,2^. For many types of pathologies, the disease process can have a wide continuous spectrum of severities, which can change over time. However, these grades of disease severity are usually binned into ordinal classes (e.g., normal, mild, moderate, severe), and changes within these ordinal classes may not be appreciated. This is further confounded by the substantial variability in the interpretation of classes by domain experts, which can lead to changes in clinical management^3,4^. Thus, grading disease severity and evaluating change on a continuous spectrum has the potential to add value to patient care and clinical research.

Deep learning is a powerful approach for automating tasks within medical imaging^5,6^. However, most of the published literature has focused on the prediction of absolute labels, with binning of patient images into discrete categories. Predicting the absolute class labels for two images and comparing them is one approach to evaluating disease severity and change, but granularity beyond the discrete class labels is lost. Thus, this strategy does not reflect the true continuous spectrum of change^4^. The lack of reliable standard image labels is also a barrier to the training and evaluation of these algorithms^7^.

**Fig. 1:**
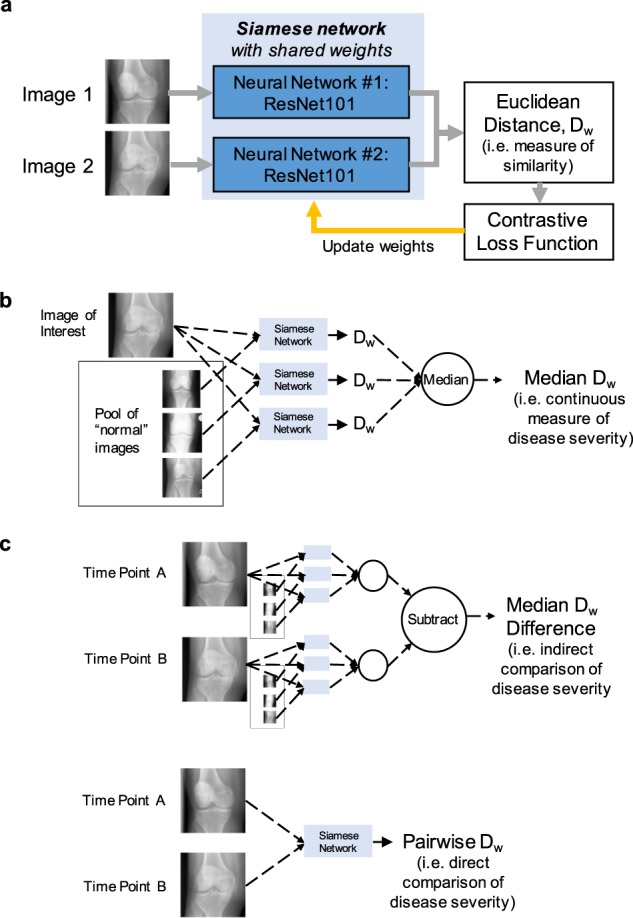
Schematics of Siamese neural network approaches for evaluating disease severity and change on a continuous spectrum. **a** Schematic of the Siamese neural network architecture, which takes two images as inputs and outputs the Euclidean distance between the two images (i.e., a measure of similarity). **b** Schematic of evaluating a single image for disease severity on a continuous spectrum. **c** Schematic of two approaches for evaluating longitudinal images for disease severity on a continuous spectrum. *D*_w_ refers to the Euclidean distance.

Evaluating the difference between imaging studies can be reformulated as a distance metric-learning problem^8^. In this subfield of machine learning, algorithms have been developed to evaluate the similarity (or dissimilarity) of data. One approach to evaluating the similarity between two images is the Siamese neural network, which was initially developed to verify the authenticity of credit card signatures^9^. Prior healthcare-related studies have used Siamese neural networks to evaluate patient similarity in the electronic health record^10^ and to help predict symptom trajectories in Alzheimer’s patients using data from multiple time points^11^. While neither of these studies used actual images as inputs, the Siamese neural network inputs can consist of paired images, with each image passed through an identical deep-convolutional subnetwork. This type of algorithm can be trained using a contrastive loss function^12^. A measure of image similarity can be obtained by calculating the Euclidean distance between the twinned subnetwork outputs (schematic in Fig. 1a). In principle, the larger the Euclidean distance, the larger the difference between the images with respect to the image features for which the network is training. Thus, we hypothesized that the continuous spectrum of disease severity and change in medical imaging can be abstracted to the Euclidean distance calculated from a Siamese neural network.

In this study, we use two data sets that contain medical images for patients followed longitudinally, annotated with ordinal classifications of disease severity. These include retinal photographs evaluating ROP (from the Imaging and Informatics in Retinopathy of Prematurity (i-ROP) cohort study^13^, 4861 images from 870 patients) and knee radiographs evaluating osteoarthritis (from the Multicenter Osteoarthritis Study (MOST), 10,012 images from 3021 patients). ROP is a leading cause of childhood blindness globally, and management strategies depend on the diagnosis of plus disease, a ROP classification, and its precursor, pre-plus disease via fundoscopic examination^14,15^. Knee osteoarthritis is a common cause of morbidity worldwide and can be diagnosed by radiography, which also can help identify which patients may benefit from surgery^16^. Automated diagnosis and classification of both of these diseases have been reported using deep-learning approaches^13,17^, but the change of disease severity (which is often more granular than the disease classification) is also relevant to clinical decision making. In this study, we use a Siamese neural network approach to represent the continuous spectra of disease severity and longitudinal change on retinal photographs for ROP and on knee radiographs for osteoarthritis.

## Results

### Siamese neural network outputs correlate with disease severity on a continuous spectrum

A convolutional Siamese neural network was built using two identical ResNet-101^18^ subnetworks, with a contrastive loss function (schematic in Fig. 1a; see Methods for details)^12^. The Siamese neural network architecture was applied in two clinical use cases involving ordinal categories of disease severity that can change over time, first for ROP and second for knee osteoarthritis. In the first case, each input image was a retinal photograph for a single eye, with the retinal vessels segmented using a previously published algorithm^13^. In the second case, most knee radiographs contained bilateral knees; thus, we cropped the images for the individual left and right knees using a previously published algorithm^19^, which were then used for the input images. In both cases, we trained the Siamese neural network on paired images (from any patient in the training set), with the binary label of change or no change in disease severity category. A validation set was used to select the best model. There is no overlap in patients between the training, validation, and test sets, as the data sets had been randomly partitioned to contain 80%, 10%, and 10% of patients, respectively.

We tested the trained networks on a 100-image subset of the ROP test set and a 100-image subset of the knee osteoarthritis test set, which were both annotated by multiple expert clinicians for disease severity ranking. The disease severity ranking was based on a previously published method that incorporates the expert comparison labels of all image pair possibilities (i.e., less versus more severe disease)^20^. For ROP, we used a previously published disease severity ranking on the 100-image subset^13^. For knee osteoarthritis, three radiologists (two fellowship-trained musculoskeletal radiologists and one senior radiology resident) manually annotated the subset to generate a disease severity ranking. The top five images with the least severe disease (i.e., most normal) were used as anchor images to which all the other images were compared using the Siamese neural network (schematic in Fig. 1b). For each image, the median of the Euclidean distances relative to the anchor images was calculated.

**Fig. 2:**
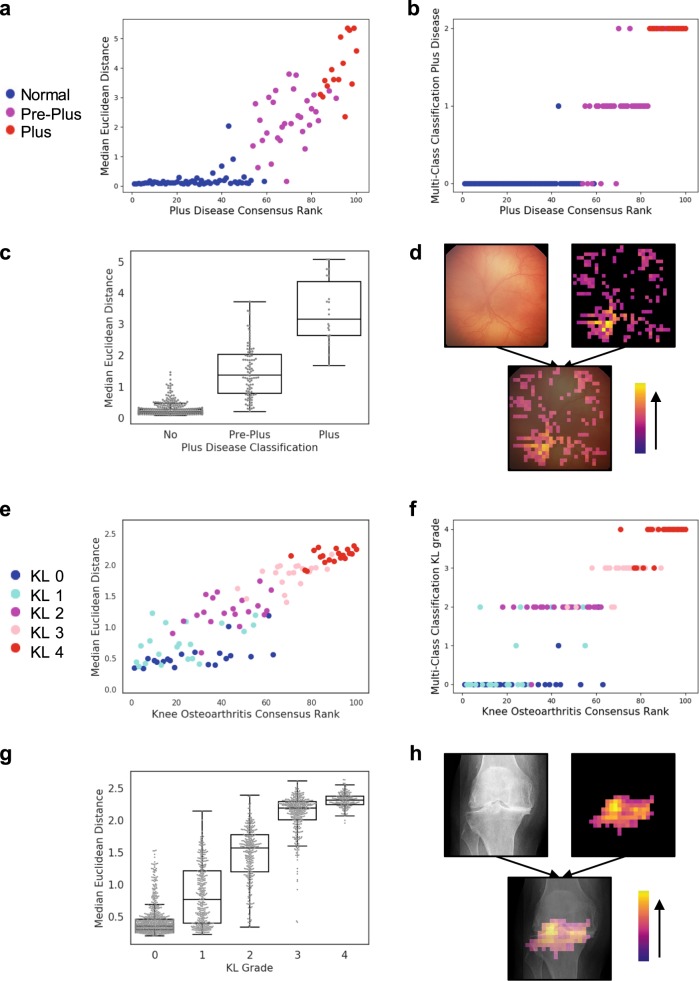
Siamese neural network outputs can be used to represent a continuous spectrum of disease severity. **a** Scatterplot shows the median Euclidean distance versus consensus disease severity rank for 100 retinal photographs ranked by experts for severity of retinopathy of prematurity (plus disease classification). The five retinal photographs with the least severe disease (i.e., most normal) were used as the anchor images to which all the other images were compared. **b** Scatterplot shows the output of a conventional neural network trained for multi-class classification of plus disease severity. *y*-axis 0, 1, and 2 indicate ordinal plus disease severity grades, corresponding to normal, pre-plus, and plus disease. **c** Boxplot* shows the median Euclidean distance calculated in relation to ten randomly sampled “normal” images, separated by plus disease classification. **d** Illustrative example of an occlusion sensitivity map for visualization of salient areas of the image. **e** Scatterplot shows the median Euclidean distance versus consensus disease severity rank for 100 knee radiographs ranked by experts for severity of knee osteoarthritis (KL grade). The five knee radiographs with the least severe disease (i.e., most normal) were used as the anchor image to which all the other images were compared. **f** Scatterplot shows the output of a conventional neural network trained for multi-class classification of KL grade. **g** Boxplot* shows the median Euclidean distance calculated in relation to ten randomly sampled “normal” images versus the KL grade. **h** Illustrative example of an occlusion sensitivity map for visualization of salient areas of the image. *Boxplot boxes indicate the median and interquartile range (IQR), with whiskers extending to points within 1.5 IQRs of the IQR boundaries.

This median Euclidean distance correlates with disease severity rank for both ROP (*ρ* = 0.87) (Fig. 2a) and knee osteoarthritis (*ρ* = 0.89) (Fig. 2e). In the case of ROP, there is a very shallow slope for images categorized as normal, as compared to the images categorized as pre-plus or plus disease (Fig. 2a). Within these discrete disease categories, there is also a correlation between the Euclidean distance and disease severity rank (*ρ* = 0.41, 0.36, and 0.60 for normal, pre-plus, and plus disease, respectively). Similarly, in the case of knee osteoarthritis, there is a correlation between the Euclidean distance and disease severity rank within the discrete disease categories (*ρ* = 0.65, 0.41, 0.51, 0.45, and 0.56 for Kellgren-Lawrence (KL) grades 0, 1, 2, 3, and 4, respectively). These findings show how the Euclidean distance of the Siamese neural network can provide a representation of disease severity on a continuous spectrum, more granular than the ordinal disease categories. This can be contrasted with the discrete output of a conventional deep-convolutional neural network, trained for multi-class classification (Fig. 2b, f).

In the absence of disease severity ranking labels, for any given image, a Siamese neural network median Euclidean distance can also be calculated relative to a pool of randomly sampled “normal” images (schematic in Fig. 1b). This median Euclidean distance correlates with the ordinal disease severity category, as demonstrated in the separate test sets for ROP (*ρ* = 0.69, *N* = 528) (Fig. 2c) and knee osteoarthritis (*ρ* = 0.87, *N* = 1908) (Fig. 2g). The location of the disease on the image can be visualized using an occlusion sensitivity map-based approach (illustrative examples in Fig. 2d, h).

### Siamese neural network outputs correlate with longitudinal change in disease severity

**Fig. 3:**
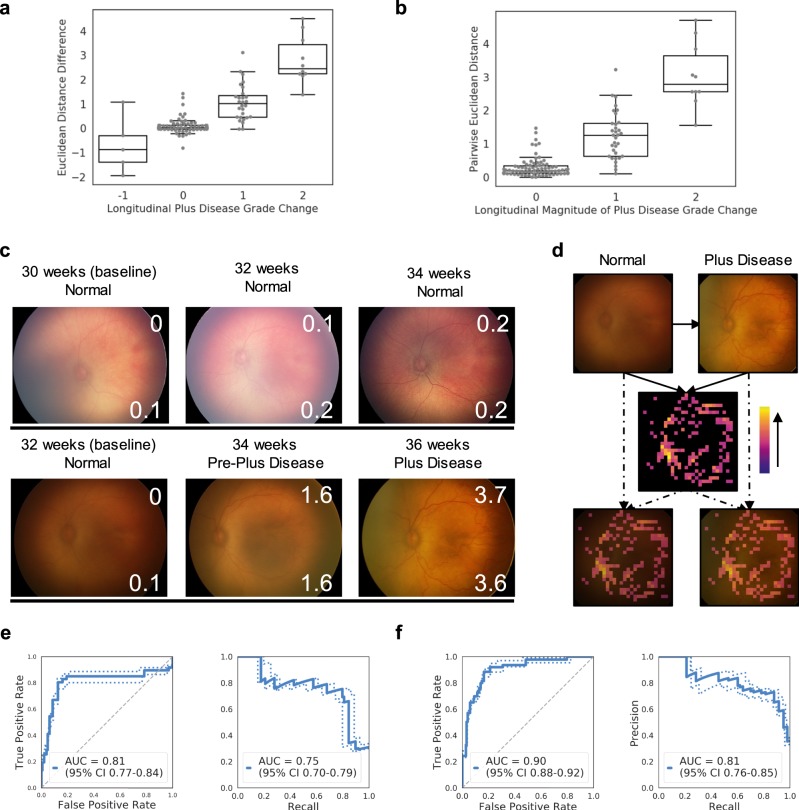
Siamese neural network outputs can be used to represent a continuous spectrum of longitudinal change in disease severity, as illustrated with retinopathy of prematurity (plus disease classification). **a** Boxplot* shows the median Euclidean distance difference between two different time points versus the longitudinal change in plus disease grade. **b** Boxplot* shows the pairwise Euclidean distance from direct comparison of two images versus the magnitude of longitudinal change in plus disease grade. **c** Demonstrative examples of longitudinal tracking of disease severity using Euclidean distances on retinal photographs. The number of weeks annotating the photographs indicate the neonatal post menstrual age. In each image, the top right inset number is the pairwise Euclidean distance between that image and the baseline image. The bottom right inset number is the median Euclidean distance relative to a pool of ten “normal” images. **d** Illustrative example of an occlusion sensitivity map for visualization of salient areas of longitudinal change between two images from the same patient (using pairwise Euclidean distance). **e** ROC and precision-recall curves for the evaluation of plus disease change from normal to pre-plus or plus disease on a separate test set, using the median Euclidean distance difference as the continuous metric for change. **f** ROC and precision-recall curves for the evaluation of plus disease change from normal to pre-plus or plus disease on a separate test set, using the pairwise Euclidean distance as the continuous metric for change. *Boxplot boxes indicate the median and interquartile range (IQR), with whiskers extending to points within 1.5 IQRs of the IQR boundaries.

**Fig. 4:**
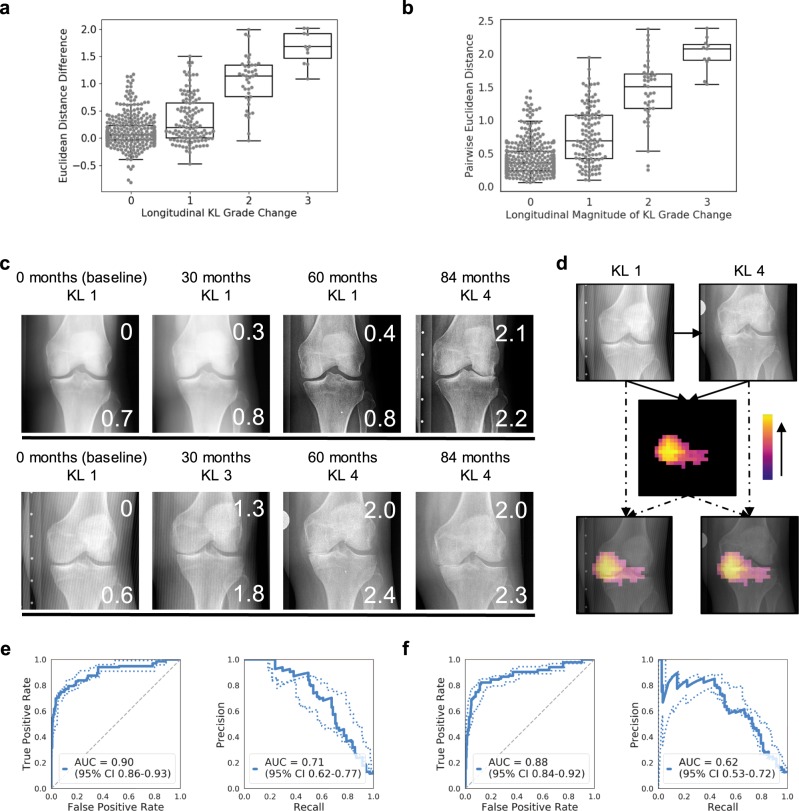
Siamese neural network outputs can be used to represent a continuous spectrum of longitudinal change in disease severity, as illustrated with knee osteoarthritis (KL grade). **a** Boxplot* shows the median Euclidean distance difference between two different time points versus the longitudinal change in KL grade. **b** Boxplot* shows the pairwise Euclidean distance from direct comparison of two images versus the magnitude of longitudinal change in KL grade. **c** Demonstrative examples of longitudinal tracking of disease severity using Euclidean distances on knee radiographs. In each image, the top right inset number is the pairwise Euclidean distance between that image and the baseline image. The bottom right inset number is the median Euclidean distance relative to a pool of ten “normal” images. **d** Illustrative example of an occlusion sensitivity map for visualization of salient areas of longitudinal change between two images from the same patient (using pairwise Euclidean distance). **e** ROC and precision-recall curves for the evaluation of plus disease change from normal to pre-plus or plus disease on a separate test set, using the median Euclidean distance difference as the continuous metric for change. **f** ROC and precision-recall curves for the evaluation of plus disease change from normal to pre-plus or plus disease on a separate test set, using the pairwise Euclidean distance as the continuous metric for change. *Boxplot boxes indicate the median and interquartile range (IQR), with whiskers extending to points within 1.5 IQRs of the IQR boundaries.

The median Euclidean distance calculated relative to a pool of randomly sampled “normal” images can also be used to assess the change in disease severity over time, by subtracting the calculated Euclidean distance at one time point from another (schematic in Fig. 1c). We created longitudinal image comparison test sets, featuring paired images from two different points in time for each test patient. In these test sets, there is a correlation between the size of the Euclidean distance difference and the change in disease severity grade for both ROP (*ρ* = 0.72) (Fig. 3a) and knee osteoarthritis (*ρ* = 0.44) (Fig. 4a). For 38 longitudinal test comparisons of retinae with a plus disease classification change of ≥1 ordinal class, 36 (95%) showed a Euclidean distance difference >0. Conversely, for five test comparisons with a plus disease classification change of ≤ –1, 4 (80%) showed a Euclidean distance difference <0. For 176 longitudinal test comparisons of knees with a KL grade change of ≥1, 145 (82%) showed a Euclidean distance difference >0. For 52 test comparisons with a KL change grade of ≥2, 51 (98%) showed a Euclidean distance difference >0. These findings show that the Euclidean distance difference usually maintains the rank order in severity between longitudinal images, while also accounting for the direction of change. KL grade monotonically increases, so evaluation of a negative KL grade change is precluded.

An alternative approach to evaluating change between two images from the same patient acquired at different time points is to directly compare the images using the Siamese neural network, calculating the pairwise Euclidean distance between the two images (schematic in Fig. 1c). While this approach loses the directionality of change in severity that the prior approach shows, the only images required are the two images being compared. In the longitudinal comparison test sets, there is a correlation between the size of the pairwise Euclidean distance and the magnitude of change in disease severity grade for both ROP (*ρ* = 0.74) (Fig. 3b) and knee osteoarthritis (*ρ* = 0.55) (Fig. 4b). Using this direct comparison approach, the location of change between the two images can be visualized using an occlusion sensitivity map-based approach (illustrative examples in Figs. 3d and 4d).

Both the Euclidean distance difference (in relation to a pool of “normal” images) and pairwise Euclidean distance approaches for evaluating longitudinal change between images can be useful for tracking change over multiple time points (illustrative examples in Figs. 3c and 4c).

### Performance on binary change detection tasks

We further evaluated the performance of the Siamese neural network for binary change detection tasks, using the longitudinal change comparison test set. For the ROP data, we tested the detection of binary change between normal and abnormal (pre-plus or plus disease) retinal photographs. For the knee osteoarthritis data, we tested the detection of binary change between normal to minimal disease (KL grades 0 or 1) and mild to severe disease (KL grades 2, 3, or 4). Receiver operator characteristic curves and precision-recall curves were generated using either the Euclidean distance difference or the pairwise Euclidean distance as the continuous prediction score. The binary change thresholds were determined by Youden’s J statistic on the validation set. The network trained for ROP achieved an AUC of 0.81 (95% CI 0.77–0.84) with Cohen’s kappa 0.64 (95% CI 0.58–0.68) and an AUC of 0.90 (95% CI 0.88–0.92) with Cohen’s kappa 0.66 (95% CI 0.61–0.71) using the Euclidean distance difference or pairwise Euclidean distance for prediction, respectively (Fig. 3e, f). The network trained for knee osteoarthritis achieved an AUC of 0.90 (95% CI 0.86–0.83) with Cohen’s kappa 0.47 (95% CI 0.40–0.54) and an AUC of 0.88 (95% CI 0.84–0.92) with Cohen’s kappa 0.41 (95% CI 0.35–0.48), using the Euclidean distance difference or pairwise Euclidean distance for prediction, respectively (Fig. 4e, f). Precision-recall curves are also shown in these figures. Performance using either the Euclidean distance difference or the pairwise Euclidean distance for the continuous prediction score was similar.

The performance on these binary change detection tasks was compared to a conventional convolutional neural network (using the same underlying ResNet-101 architecture as the Siamese neural network), trained using cross-entropy loss for multi-class classification of the ordinal disease categories. For the binary change detection tasks, change was determined using the maximum probability label assigned by the neural network. For ROP, the conventional neural network achieved a linear Kappa of 0.61 (95% CI 0.55–0.66). For knee osteoarthritis, the conventional neural network achieved a linear Kappa of 0.46 (95% CI 0.39–0.54). Given the overlap in the 95% confidence intervals with the Siamese neural network determined Kappa values, the performance of the Siamese neural network on a binary change detection task is similar compared to a conventional classification network.

## Discussion

We leveraged a Siamese neural network architecture to evaluate disease severity and change on a continuous spectrum, as illustrated in two applications within ophthalmology and radiology. The Euclidean distance between the final fully connected layers of the twinned Siamese neural network subnetworks can be used to provide a meaningful measures of disease severity relative to normal cases or other time points in the same patient, which supports our hypothesis. Importantly, we show that continuous disease severity and change evaluation can be performed without specific localization of the pathology of interest (such as abnormal joint space narrowing or tortuous vessels), requiring only image-level annotation. In addition, only binary comparison labels are required to train the network (i.e., same versus different). The work shown has potential application to any disease that involves a continuous spectrum of severity, with particular utility in conditions where patients are imaged at multiple time points.

The discrete binning of disease classes is human-engineered and the underlying biology of disease is usually more accurately described as a spectrum^4^. In addition, previous work has shown that there is substantial intra- and inter-expert variability in the annotation of both plus disease classification and KL grade^4,21,22^. As an alternative to discrete clinical grading schemes like these or the non-standard ordinal designations assigned frequently in clinical practice (e.g., mild, moderate, severe), we envision a future where continuous disease severity scores can be used to assess disease severity and track change over time in a more consistent fashion. This could be helpful in both routine clinical care and clinical research. Previous work on KL grade classification using deep learning has explored the utility of using a continuous metric for performance evaluation (regression loss) to improve model accuracy^23^, but that classification system ultimately results in the prediction of ordinal disease grades. Our Siamese neural network approach, using measures derived from the Euclidean distance output, provides a potentially generalizable solution for creating continuous disease severity grading scales for medical images.

An interesting property of the Siamese neural network is that the model requires only binary comparison labels for training, but still implicitly learns the magnitude of difference in disease severity between the two input images. This can decrease the annotation burden for clinical experts annotating training data, as annotating a binary difference between two images is usually an easier task than categorizing the disease severity on a single image. As interrater reliability has been shown to be higher for disease severity rankings than the classification of ordinal disease categories^20^, this approach could also decrease the noise in training labels, which could potentially reduce the amount of data needed to train the model.

A potential concern regarding the output of a Siamese neural network for evaluating differences between two images is that, instead of learning disease severity features, the network is learning features related to other patient differences or image technique. Training these algorithms using between-patient comparisons helps to mitigate the impact of such confounders. For example, in Fig. 2a, e, each different patient image is compared to a pool of “normal” images. If the network primarily learned features related to other patient differences or image technique, the median Euclidean distance would be randomly distributed in relation to expert disease severity rank. However, we found a strong correlation, showing that the network is indeed learning to represent disease severity. The occlusion sensitivity maps further illustrate that the algorithms focus on relevant disease image features.

There are several limitations to this work. First, the labels used for training were derived from the ordinal disease severity class labels. Future studies may incorporate labels assigned by expert comparisons of images for training instead. In addition to possibly improving the performance of training, the comparison labels may be incorporated with ordinal disease severity class labels for training to potentially decrease the number of images that need to be annotated^24^. Second, we demonstrated the utility of this approach for two-dimensional imaging problems, while much medical imaging involves three-dimensional data. Future studies will need to investigate the extensibility of this approach to volumes. Third, the models trained in this study utilized a contrastive loss function applied to the Euclidean distance calculated from the last fully connected layers. Additional approaches, such as testing of the loss function on earlier network layers or other loss functions entirely such as the triplet loss function, margin rank loss, and other alterations to the training methodology may further improve performance of the models^25^. Other loss functions may also incorporate more information in the labels, such as magnitude and directionality, which could potentially further improve learning.

## Methods

The parts of the study involving neonatal retinal image data were performed as a part of the multicenter i-ROP consortium, with approval by the Institutional Review Board at the coordinating center (Oregon Health & Science University), and by each of the eight participating institutions (Columbia University, Cornell University, University of Illinois at Chicago, William Beaumont Hospital, Children’s Hospital Los Angeles, Cedars-Sinai Medical Center, University of Miami, and Asociación para Evitar la Ceguera en México). This study was done in accordance with the Declaration of Helsinki, and written informed consent was previously obtained from parents of all infants enrolled. The parts of the study involving knee radiographs used de-identified publicly available data from the Multicenter Osteoarthritis Study (MOST).

### Imaging data sets

Posterior retinal photographs evaluating ROP were acquired from the Imaging and Informatics in Retinopathy of Prematurity (i-ROP) cohort study^13^, of which there were 4861 images from 870 untreated patients. Separate images were acquired for each eye. These photographs were previously classified into three clinical categories (normal, pre-plus disease, or plus disease, in order of increasing disease severity) using a reference standard diagnosis based on a consensus of image grading method^26^. These patients had been imaged at varying time points as clinically indicated, ranging from a post menstrual neonatal age of 30 to 98 weeks. The majority of the retinal photographs were classified as normal (*N* = 4097), as opposed to pre-plus disease (*N* = 635) or plus disease (*N* = 129).

Posteroanterior (PA) weight-bearing bilateral knee radiographs evaluating knee osteoarthritis were acquired from the Multicenter Osteoarthritis Study (MOST), of which there were 10,012 images from 3021 patients with longitudinal radiographic follow-up. This data set contains KL grades (0, 1, 2, 3, or 4, in order of increasing knee osteoarthritis disease severity), assigned by expert readers to each knee. Knee images assigned the non-standard KL grade of 1.9 in the MOST data set were omitted from this analysis. All patients in the data set had baseline imaging data, and varying numbers of patients were imaged again at 15, 30, 60, and 84 months. The KL grade for each knee was distributed as follows (noting that some images showed only one knee instead of two): 0 (*N* = 7704), 1 (*N* = 2862), 2 (*N* = 3194), 3 (*N* = 3558), and 4 (*N* = 1714).

Both data sets were randomly partitioned at the patient level into algorithm training, validation, and testing sets, containing 80%, 10%, and 10% of patients, respectively. An additional test data set of 100 retinal photographs from 100 unique patients was also reserved from the i-ROP cohort study (normal *N* = 54, pre-plus disease *N* = 31, plus disease *N* = 15). These images had been previously compared to each other and ranked by disease severity by a consensus of multiple expert raters^20^. From the knee osteoarthritis test set, 20 radiographs were randomly sampled from each KL grade category, yielding 100 total images. These images were compared to each other and ranked by disease severity by a consensus of multiple raters (two radiologists with musculoskeletal imaging sub-specialty training and one radiology resident), using the same method as previously described for the retinal photographs^20^. The raters were presented with 5446 total pairwise image comparisons derived from the 100 total images (with 10% of comparisons as repeats), which were manually annotated as the same or different.

### Longitudinal comparison test set creation

In the test sets, patients have images from a varying numbers of time points. Sampling all possible longitudinal image combinations would skew the test comparisons towards patients with images from more time points. Thus, to create longitudinal comparison test sets, we sampled the test sets so that each unique patient knee or retina is only tested once. Given that many fewer patients showed a longitudinal change compared to those with no change between two images, if a combination with change was possible, that image combination was used. If multiple combinations with change were possible, then one of those image combinations was randomly sampled. If no change combinations were possible, then any image combination was randomly sampled. This approach resulted in longitudinal comparison test sets of 123 comparisons from 65 patients for ROP (80 with no change in plus disease classification and 43 with a change) and 521 comparisons from 272 patients for knee osteoarthritis (345 with no change in KL grade and 176 with a change).

### Image pre-processing

Retinal photographs from the i-ROP cohort study are of variable sizes and types; thus, they were all resized to 300 × 225 pixels (maintaining the aspect ratio) and converted to portable network graphic (PNG) files. As the retinal vessels are the structures of interest, we used a previously published U-Net convolutional neural network architecture to segment the vessels, thereby removing background variation^13^. For training, images were augmented using random rotation (range of ±10° of rotation), horizontal flipping (50% probability), random pixel crops (to 224 × 224 pixels), and random brightness and contrast variation (range of ±3%). For validation and testing, images were center cropped (to 224 × 224 pixels). Color images were converted to greyscale in all cases.

Knee radiographs from the MOST data set mostly contain bilateral knees. We used a previously published machine-learning approach to localize the knee joint areas^19^, which outputted 140 × 140 mm bounding boxes (resized to 224 × 224 pixels). All images were processed with global contrast normalization. Left knees were vertically flipped so that all knees would appear as right knees. For training, images were augmented using random rotation (range of ± 5° of rotation), random pixel crops (to 210 × 210 pixels), and random brightness and contrast variation (range of ±5%). For validation and testing, images were not augmented. Color images were converted to greyscale in all cases.

### Siamese neural network architecture and training strategy

Siamese neural networks take two separate images as inputs, and pass them through identical neural subnetworks, which are joined at the output^9^. A convolutional Siamese neural network was built using two identical ResNet-101^18^ subnetworks with shared weights, pre-trained on the ImageNet data set for increased stability of training^27^. ResNet-101 was selected for its optimal performance after empirically testing ResNets-18, 34, 50, 101, and 152. The final fully connected layer of ResNet-101 was modified to output three or five nodes from both subnetworks, for the retina and knee algorithms, respectively (~42.5 × 10^6^ total parameters in both models). Given image input vectors X_1_ and X_2_, the Euclidean distance *D*_w_ between the subnetwork outputs, *G*_w_(X_1_) and *G*_w_(X_2_), can be calculated $\left( {D_{\mathrm{w}}\left( {X_1,X_2} \right) = ||G_{\mathrm{w}}\left( {X_1} \right) - G_{\mathrm{w}}\left( {X_2} \right)||_2} \right)$^12^. The neural network parameters were trained using the contrastive function $\left( {L = \left( {1 - Y} \right)D_{\mathrm{w}}^2 + \left( Y \right)\{ {\mathrm{max}}\left( {0,m - D_{\mathrm{w}}} \right)\} ^2} \right.$ ; *Y* = 0 if same class (i.e., no change) and *Y* = 1 if different class (i.e., change), and *m* = margin)^12^. The contrastive loss function is minimized if there is a small *D*_w_ for no change and large *D*_w_ for change. The margin was empirically set to 2.0 and indicates the maximum *D*_w_ at which dissimilar paired inputs will not contribute further to the loss, which helps to stabilize the loss function in training. Implementation of this Siamese network architecture was performed using the Python package PyTorch, using the Adam optimizer^28^ (initial learning rate = 0.000005, *β*_1_ = 0.9, *β*_2_ = 0.999), batch sizes of 16 for training and validation, and early stopping of training when the validation loss showed no further improvement after three training epochs. The network parameters from the model with the lowest validation loss were saved for testing evaluation.

For both data sets, we trained a Siamese neural network on paired input images sampled from from different patients and time points (i.e., both inter-patient and intra-patient comparisons). Because of the class asymmetries in the i-ROP cohort study data, we randomly sampled an equal number of paired input retinal photographs with the same or different plus disease classification for training and validation. Similarly, for training and validation for the MOST data set, paired input knee joint images (localized to the knee joint as described above) were randomly sampled for an equal distribution of pairs with the same or different KL grade.

The number of image pairs sampled per epoch of training was optimized empirically for both data sets, with saturation of validation loss using 3200 and 32,000 paired comparisons for the i-ROP and MOST data sets, respectively. The number of image pairs sampled for validation was 1600 and 6400, respectively.

### Conventional multi-class classification neural network training strategy

A multi-class classification network for ordinal disease severity category was created, sharing the same underlying ResNet-101^18^ architecture as the Siamese neural network described above. As done with the Siamese neural network, the final fully connected layer of ResNet-101 was modified to output three or five nodes, for the retina and knee algorithms, respectively (~42.5 × 10^6^ total parameters in both models), to match the number of ordinal disease severity classes. These nodes provided the input for the softmax function. The maximum probability label was used to assign class labels. The network parameters were trained using the cross-entropy loss function. This network architecture was implemented using the Python package PyTorch, using the Adam optimizer^28^ (initial learning rate = 0.000005, *β*_1_ = 0.9, *β*_2_ = 0.999), batch sizes of 16 for training and validation, and early stopping of training when the validation loss showed no further improvement after three training epochs.

### Evaluation of single-image disease severity

To evaluate a single image for its disease severity on a continuous spectrum, the image is compared to a pool of ten randomly sampled “normal” images (i.e., normal retinal photographs and KL grade 0 knee radiographs). Using the Siamese neural network, the Euclidean distance is calculated for each paired input of the image of interest and the ten “normal” images. The median of these ten Euclidean distances is computed, which serves as the Euclidean distance used as a measure of disease severity. For the 100-image test sets annotated by multiple experts for disease severity ranking, a Euclidean distance is calculated from the paired input of each image and each of the five images with the lowest disease severity ranking. The median of these five Euclidean distances is computed to give a measure of disease severity.

### Evaluation of longitudinal disease severity change

To evaluate two images from the same patient for the change in disease severity on a continuous spectrum, we used two approaches. First, the Euclidean distance for each image can be calculated from the single-image disease severity evaluation approach described above. The difference between these Euclidean distances can be used to represent change between the two images. Second, a Euclidean distance can be calculated by directly inputting the two images of interest to the Siamese neural network. This Euclidean distance provides another representation of change between the two images.

### Neural network visualization using occlusion sensitivity

We used an occlusion sensitivity approach to visualize what areas of the paired images were important for the determination of the Euclidean distance^29^. In this approach, patches of *N* x *N* pixels (*N* = 8 for retinal photographs and *N* = 32 for knee radiographs) in both of the paired images are occluded (patch area pixel intensities set to the mean of the patch), which occurs iteratively across the entire image (stride length of 8 pixels). The patch sizes were selected empirically based on the tradeoff between spatial sensitivity and noise. For each iteration, both occluded images are passed through the Siamese neural network and a Euclidean distance is calculated. The difference is taken between this Euclidean distance and the non-occluded baseline Euclidean distance. This difference increases when a patch occludes an area in the image that is relevant to the Siamese neural network, which can be represented as heat map. When evaluating disease severity in a single image as described above, an occlusion sensitivity map is generated for each comparison of the image of interest to an image from the pooled “normal” images. The median of these occlusion sensitivity maps is used for visualization. When evaluating longitudinal disease severity change as described above, an occlusion sensitivity map is generated using the two input images, which is used for visualization.

### Data analysis

Associations between the Siamese neural network outputs and disease severity ranking or ordinal disease severity class or class change were calculated using Spearman’s rank correlation (*ρ*). For the binary change detection tasks, receiving operator characteristic (ROC) and precision-recall AUCs were calculated on the testing data using Euclidean distances. Bootstrap 95% confidence intervals were calculated for each AUC. For a binary label assignment by the Siamese neural network, a Euclidean distance threshold is calculated by taking the Euclidean distance with the maximum Youden’s J statistic in the validation set. Linear Cohen’s Kappa coefficients were calculated for binary change predictions, also with bootstrap 95% confidence intervals. Data visualizations were implemented using the Matplotlib, OpenCV, and Seaborn Python packages.

### Reporting summary

Further information on research design is available in the Nature Research Reporting Summary linked to this article.

## Supplementary information

Supplementary Information

Reporting Summary

## Data Availability

The i-ROP cohort study data for ROP is not publicly available due to patient privacy restrictions, though potential collaborators are directed to contact the study investigators (https://i-rop.github.io/). The MOST data set for knee osteoarthritis is made publicly available from the MOST study investigators and can be requested from them (http://most.ucsf.edu/).

## Code availability

The key code used for this study is available at https://github.com/QTIM-Lab/SiameseChange.
