# Supplementary material for: Siamese neural networks for continuous disease severity evaluation and change detection in medical imaging
Source: NPJ Digit Med. 2020 Mar 26;3:48. doi: 10.1038/s41746-020-0255-1 (PMC7099081; doi:10.1038/s41746-020-0255-1)
Supplement: Supplementary file 1 — Supplementary Information [file 41746_2020_255_MOESM1_ESM.pdf]

Supplementary Notes

i-ROP research consortium

MEMBER LIST (in alphabetical order)

Andrea Arriola[8], Andrew Beers[1], Audina Berrocal[9], J. Peter Campbell[3], RV Paul Chan[10], Ken Chang[1], Tammy Check[11], Ida Chen[12], Michael F. Chiang[3], Kristi Cumming[11], Osode Coki[13], Kimberly Denser[11], Carlos M. Dulanto-Reinoso[8], Cheryl-Ann Eccles[13], Deniz Erdoğmuş[5], Sharon Galvis[14], Jason Horowitz[13], Stratis Ioannidis[5], Karyn Jonas[10], Jayashree Kalpathy-Cramer[1], Sang Jin Kim[3], Evan Kruger[15], Thomas Lee[15], Xiaohui Li[12], Maria Ana Martinez-Castellanos[8], Miroslava Meraz-Gutierrez[8], Kathryn McGovern[15], Cristina Montero-Mendoza[8], Raghu Murthy[14], Catherin Negron[9], Francisco Olguin-Manriquez[8], Anton Orlin[16], Tammy Osentoski[11], Susan Ostmo[3], Kaye Roll[12], Rafael Romero[8], Jerome Rotter[12], Samantha Salinas-Longoria[8], Leora Sarna[13], Charles Simmons[14], Kemal Sonmez[3], Kent Taylor[12], Mary Zajechowski[11]

UNIQUE AFFILIATIONS

8. Asociacion para Evitar la Ceguera en Mexico, Mexico City, Mexico
9. Bascom Palmer Eye Institute, Miami, FL, USA
10. University of Illinois at Chicago, Chicago, IL, USA
11. William Beaumont Hospital, Royal Oak, MI, USA
12. LA Biomedical Research Institute, Los Angeles, CA, USA
13. Columbia University, New York, NY, USA
14. Cedars Sinai Hospital, Los Angeles, CA, USA
15. Children's Hospital Los Angeles, Los Angeles, CA, USA
16. Weill Cornell Medical College, New York, NY, USA
